# No Effect of Omega-3 Fatty Acid Supplementation on Cognition and Mood in Individuals with Cognitive Impairment and Probable Alzheimer’s Disease: A Randomised Controlled Trial

**DOI:** 10.3390/ijms161024600

**Published:** 2015-10-16

**Authors:** Michelle A. Phillips, Caroline E. Childs, Philip C. Calder, Peter J. Rogers

**Affiliations:** 1 School of Experimental Psychology, University of Bristol, Bristol BS8 1TU, UK; E-Mail: peter.rogers@bristol.ac.uk; 2 Human Development and Health Academic Unit, Faculty of Medicine, University of Southampton, Southampton SO16 6YD, UK; E-Mails: cch@mrc.soton.ac.uk (C.E.C.); pcc@soton.ac.uk (P.C.C.); 3 NIHR Southampton Biomedical Research Centre, University Hospital Southampton NHS Foundation Trust and University of Southampton, Southampton SO16 6YD, UK

**Keywords:** Alzheimer’s disease (AD), cognitive impairment no dementia (CIND), omega-3 fatty acids, eicosapentaenoic acid (EPA), docosahexaenoic acid (DHA)

## Abstract

Findings from epidemiological and observational studies have indicated that diets high in omega-3 polyunsaturated fatty acids (PUFAs) such as docosahexaenoic acid (DHA) and eicosapentaenoic acid (EPA) may reduce the risk of cognitive decline and Alzheimer’s disease (AD). To determine if increasing intake of DHA and EPA through supplementation is beneficial to cognition and mood in individuals with cognitive impairment no dementia (CIND) or Alzheimer’s disease (AD) a four month, randomised, double-blind, placebo controlled study was conducted. Fifty-seven participants with CIND and nineteen with AD were randomised to receive either omega-3 PUFAs (600 mg EPA and 625 mg DHA per day) or placebo (olive oil) over a four month period. Elevating depleted levels of EPA and DHA through supplementation in individuals with CIND or AD was found to have negligible beneficial effect on their cognition or mood. These findings confirm an overall negligible benefit of omega-3 PUFA supplementation for those with cognitive impairment and dementia. More intervention studies need to be undertaken with longer study durations and larger sample sizes. It may prove fruitful to examine effects of different doses as well as effects in other dementia subtypes.

## 1. Introduction

There is increasing scientific evidence from epidemiology and animal studies linking omega-3 polyunsaturated fatty acid (PUFA) intake with physical health [1,2,3,4,5]. Evidence is also being accumulated which suggests beneficial links between diets high in omega-3 PUFAs and reduced risk of cognitive decline and dementia [6]. With dementia fast becoming a global epidemic, it is clear that a major effort is needed to identify preventative and therapeutic strategies to alleviate the burden of cognitive decline and dementia. Diet, specifically omega-3 PUFAs, may beneficially modify risk factors in the aetiology of dementia [6].

Few intervention studies have investigated whether omega-3 PUFAs are beneficial to cognition in either healthy older adults or in older adults with a clinical diagnosis of dementia, specifically Alzheimer’s disease (AD) and, even less explored, cognitive impairment no dementia (CIND) [7]. The few studies that have been conducted report equivocal results. Improvements in Mini Mental State Examination (MMSE) score and quality of life were found when supplementing EPA or a combination of omega-3 and -6 PUFAs to patients with AD [8,9]. However another study contradicted these findings [10]. More recently, a larger randomised double-blind placebo controlled study, which found that supplementing DHA and EPA along with cholinesterase inhibitors to mild to moderate AD patients did not improve or delay the progression of cognitive decline [11]. However, positive effects were found in a small sub-group of mild AD patients who scored >27 on the MMSE. It has been reported that such an intervention did show positive effects on depressive and agitation symptoms in non-APOE carriers and APOE carriers, respectively [12]. This is the only published piece of work that has examined the benefits of such an intervention on the mood of individuals with AD. No published data exist about the benefits omega-3 PUFAs may have on mood in individuals with CIND, although there is a fairly large literature on effects of omega-3 PUFA supplementation in depression [13]. Moreover, there may also be potential benefits of omega-3 PUFA supplementation on cognition in individuals with mild cognitive difficulties [7]. Treatment benefits in Mild Cognitive Impairment (MCI) participants have been found but there was no benefit in AD patients [7,14,15]. There are other high quality studies published in this topic area [16,17,18,19]; however we were specifically concerned with studies recruiting individuals with clinical diagnoses of dementia of which there are still very few. Therefore, the present randomised, double-blind placebo-controlled study explored whether omega-3 PUFA supplements providing DHA and EPA benefit cognition and mood in individuals with early stage AD and CIND, rather than moderately impaired AD patients.

## 2. Results

### 2.1. Participant Characteristics

Seventy six individuals (57 CIND and 19 AD) were recruited and there were no dropouts. However, four participants (2 CIND randomised to placebo and 2 AD, one randomised to omega-3 supplements and one to placebo) did not complete all visits. Reasons for non**-**attendance were illness or hospital admission. Details of the demographic characteristics of the participants receiving omega-3 supplements or placebo are shown in Table 1. Data for individuals with CIND or with AD are combined due to the low recruitment in the latter population.

**Table 1:** Characteristics of participants in the omega-3 fatty acid and placebo groups at study entry.

Characteristic	Total Group	Omega-3 PUFA Group (*n* = 37)	Placebo Group (*n* = 39)
**Female, n (%)**	42 (55.3)	21 (56.8)	21 (53.8)
**Age, y (SD)**	71.1 (4.8)	71.1 (8.6)	71.1 (9.5)
**Education, y (SD)**	14.1 (4.8)	14.3 (5.1)	13.8 (4.5)
**NART IQ (SD)**	110 (10)	108 (9)	111 (11)
**Baseline EPA**	1.51 (1.15)	1.58 (1.31)	1.44 (0.99)
**Baseline DHA**	3.98 (1.12)	4.06 (1.14)	3.91 (1.12)

DHA, docosahexaenoic acid; EPA, eicosapentaenoic acid; n, number; NART IQ, National Adult Reading Test Intelligence Quotient; y, years.

There were no differences in age, years of education and pre-morbid (NART) IQ [20] between the two treatment groups at study entry. Participants with AD continued to receive a stable dose of a cholinesterase inhibitor for the duration of the study.

### 2.2. Compliance and Plasma Phosphatidylcholine (PC) Fatty Acids

Compliance was measured by capsule count and plasma PC fatty acid concentrations. On average both groups consumed 75.6% (SD 0.8) of their capsules.

**Figure 1:**
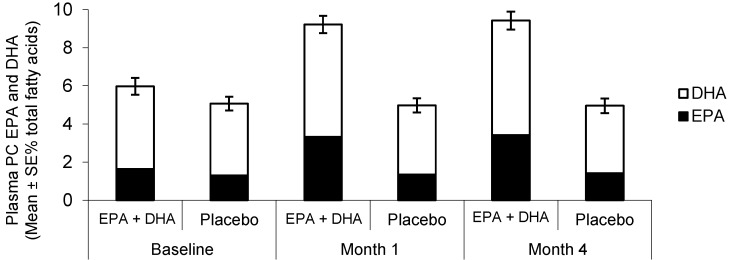
Plasma phosphatidylcholine EPA and DHA in the omega-3 and placebo groups at baseline, and after one and four months of treatment. Error bars are for total fatty acids (EPA + DHA). DHA, docosahexaenoic acid; EPA, eicosapentaenoic acid; PC, phosphatidylcholine.

Plasma PC EPA and DHA did not change in the placebo group (Figure 1). However, they both increased at month 1 in the omega-3 group (both *p* < 0.00001 *vs.* baseline, respectively) (Figure 1). There was no further increase to month 4 (Figure 1). Both EPA and DHA were higher in the omega-3 PUFA group than in the placebo group at months 1 and 4 (Figure 1). The mean increase in EPA in the omega-3 group was 137.5% and for DHA it was 38.1%.

### 2.3. Primary Outcome

There were two participants (1 CIND, 1 AD) who did not attend their month 4 appointment. In these cases, the missing data were replaced using last observation carried forward. No differences in baseline performance scores for any of the six primary outcome measures between the omega-3 PUFA and placebo groups were found (*p* > 0.158) These scores were subsequently unaffected by omega-3 PUFAs: there was no significant effect of treatment or significant treatment by month interaction for any of the outcomes (Table 2).

**Table 2:** Primary outcome measure performance scores over the study duration. Data are mean (SD). Baseline scores were included as a covariate in the analyses of the effects of treatment and treatment by month. MMSES7, mini–mental state examination Serial Sevens; MMSEWB, mini–mental state examination World Backwards; PUFA, polyunsaturated fatty acid.

Outcome Measure		Omega-3 PUFA Group	Placebo Group	*p for Main Effect of Treatment*	*p for Treatment by Month Interaction*
MMSES7	Baseline	24.3 (3.2)	23.4 (4.1)	*F* (1,73) < 1, *p* = 0.711	*F* (1,73) < 1, *p* = 0.959
	Month 1	24.4 (3.9)	23.4 (4.6)		
	Month 4	24.4 (4.1)	23.3 (4.7)		
MMSEWB	Baseline	25.0 (2.8)	24.2 (3.7)	*F* (1,73) < 1, *p* = 0.576	*F* (1,73) < 1, *p* = 0.750
	Month 1	25.3 (3.4)	24.2 (3.8)		
	Month 4	25.3 (3.4)	23.3 (4.1)		
Immediate verbal memory	Baseline	16.2 (4.3)	16.0 (5.9)	*F* (1,73) = 0.461, *p* = 0.499	*F* (1,73) = 0.567, *p* = 0.454
	Month 1	17.4 (5.1)	17.4 (6.7)		
	Month 4	16.1 (5.2)	16.7 (6.3)		
Delayed verbal memory	Baseline	3.5 (2.7)	3.1 (3.0)	*F* (1,73) < 1, *p* = 0.463	*F* (1,73) < 1, *p* = 0.998
	Month 1	3.9 (3.1)	3.9 (3.3)		
	Month 4	3.4 (2.9)	3.5 (3.2)		
Recognition verbal memory	Baseline	6.4 (3.3)	6.4 (3.6)	*F* (1,73) < 1, *p* = 0.463	*F* (1,73) = 1.67, *p* = 0.200
	Month 1	7.4 (3.8)	7.3 (3.8)		
	Month 4	7.1 (3.4)	6.3 (4.1)		
Mood	Baseline	2.7 (2.4)	2.3 (2.0)	*F* (1,73) < 1, *p* = 0.548	*F* (1,73) < 1, *p* = 0.468
	Month 1	2.3 (3.2)	2.3 (3.1)		
	Month 4	2.3 (2.9)	2.1 (2.5)		

### 2.4. Secondary Outcome Measures

There were no significant effects of treatment for any secondary outcome measures (Table 3). In addition, there were no treatment by month interactions.

**Table 3:** Secondary outcome measure performance scores over the study duration. BADLS, Bristol’s Activities of Daily Living Scale; CLOX2, clock drawing task 2; PUFA, polyunsaturated fatty acid.

Outcome Measure		Omega-3 PUFA Group	Placebo Group	*F and p Values for Main Effect of Treatment*	*F and p for Treatment by Month Interaction*
Verbal reasoning	Baseline	18.8 (6.0)	18.8 (6.4)	*F* (1,73) = 2.45, *p* = 0.122	*F* (1,73) = 1.28, *p* = 0.262
	Month 1	19.8 (5.9)	19.2 (6.7)		
	Month 4	20.1 (6.2)	18.6 (7.6)		
Visual memory	Baseline	9.72 (4.12)	9.08 (4.05)	*F* (1,73) = 2.87, *p* = 0.095	*F* (1,73) < 1, *p* = 0.863
	Month 1	10.75 (2.89)	9.41 (4.59)		
	Month 4	11.31 (4.27)	9.82 (4.07)		
CLOX2 (Executive function)	Baseline	13.8 (1.2)	13.4 (1.3)	*F* (1,73) < 1, *p* = 0.359	*F* (1,73) < 1, *p* = 0.551
	Month 1	13.8 (1.2)	14.0 (1.3)		
	Month 4	13.6 (1.8)	13.5 (1.9)		
Word finding	Baseline	13.0 (2.3)	12.4 (3.2)	*F* (1,73) = 1.15, *p* = 0.288	*F* (1,73) < 1, *p* = 0.695
	Month 1	13.4 (2.1)	12.6 (3.1)		
	Month 4	13.5 (2.5)	12.9 (2.9)		
BADLS	Baseline	2.62(5.28)	4.72 (7.34)	*F* (1,73) < 1, *p* = 0.595	*F* (1,73) < 1, *p* = 0.965
	Month 1	2.92 (5.61)	4.77 (7.11)		
	Month 4	3.35 (7.10)	5.38 (8.07)		

Data are mean (SD). Baseline scores were included as a covariate in the analyses of the effects of treatment and treatment by month.

## 3. Discussion

Although supplementation with omega-3 PUFAs raised plasma DHA and EPA concentrations, no significant treatment effects or treatment by month effects were found for any of the measures of cognitive function and mood. Among the six primary and five secondary outcomes only one effect, for visual memory, approached statistical significance. Although this was in favour of the omega-3 PUFA treatment, given the number of tests conducted, this can be regarded as a chance finding. Therefore, it can be concluded that this study found no evidence to support the suggestion that elevating omega-3 fatty acid status in individuals with CIND and early AD using omega-3 supplements has any benefit to their cognition or mood. It may also be possible to speculate that similar results would be replicated in a clinical sample of individuals with MCI. Therefore, the clinical usefulness of such an intervention is questionable.

The findings from this randomised, double-blind, placebo controlled study found that elevating omega-3 fatty acid levels in individuals with CIND and early AD does not benefit cognition or mood both support and contradict previous intervention studies in this area [7,8,9,21]. These findings support other research evidence [10,11,22] which stated that individuals with mild to moderate AD do not benefit from such an intervention. However, the findings from this study contradict other research evidence that individuals with very mild AD may benefit from omega-3 fatty acid supplements because they reduce the rate of decline in MMSE score and improve symptoms of depression and agitation [11,12]. However, the present study did not obtain data relating to whether participants were APOE4 carriers. Therefore, it is not possible to completely contradict the earlier findings which indicated that depressive symptoms were reduced by omega-3 fatty acids in non-APOE4 carriers. This study also contradicts the suggestions that this type of intervention improves cognition in those with MCI [7,13,15]. It is therefore clear that evidence remains equivocal at this time especially when the majority of intervention studies have relatively small sample sizes. Therefore, future research programmes in this area need to undertake much larger studies with larger samples sizes and longer study durations.

Although the supplement used contained similar amounts of EPA and DHA, the relative increase in the content of EPA in plasma PC from baseline was much greater than the relative increase in the content of DHA (mean increases 137.5% and 38.1%, respectively). However, this reflects an absolute increase in EPA content of 2% of fatty acids (from ~1.5% of fatty acids at baseline to ~3.5% after supplementation) and in DHA content of 1.5% of fatty acids (from ~4% of fatty acids at baseline to ~5.5% of fatty acids after supplementation). Nevertheless, EPA seems to be better incorporated than DHA into plasma PC. This conclusion is consistent with previous reports. Provision of 3 g of EPA and 3.54 g of DHA per week for one year in healthy volunteers resulted in a mean 85% relative increase in EPA content of plasma PC from baseline and a mean 44% relative increase in DHA content [23]. This greater relative uptake of EPA than DHA into plasma PC may reflect favoured metabolism of EPA into plasma PC or it may reflect preferential metabolic partitioning of DHA away from plasma PC and into other lipids.

From the results of this study it is possible to surmise that omega-3 fatty acid supplements may have a negligible benefit and thus no clinical usefulness in individuals with AD and may also not be beneficial in primary prevention and thus not useful in people with CIND. However, there are other theoretical reasons why benefits were not seen in these particular populations. Firstly, in an unpublished poster of a research project undertaken in AD mouse models the authors argued that it was possible that a study duration of four months does not give the omega-3 fatty acids enough time to show effects on amyloid accumulation, inflammatory mediators and protein expression [24]. The authors of this poster argued that omega-3 fatty acids may have reduced amyloid accumulation, inflammatory mediators and proteinase expression but these reductions may not have lead to improvements in cognition and further, may not have reached the brain so behavioural or biochemical ameliorations could not occur [24]. The poster indicated the possibility that the amyloid precursor protein (APP) expression may be so high that the omega-3 fatty acids effect may not have been detectable [24]. It is also possible that supplementation did not result in improvements to the composition of cell membranes leading to regeneration of nerve cells and thus improvements in cognition [25].

It may also be important to consider that AD is a progressive disorder where cognition is always deteriorating. Therefore, any improvements experienced as a result of intervention may be continuously overcome by the disease progression. A similar view has been argued that omega-3 fatty acids may have a role in primary prevention of AD but not in treatment of the disease once it has manifested [11]. It has also be postulated that there is a possibility that once AD is clinically evident, the neuropathologic involvement is too advanced to be attenuated substantially by anti-inflammatory treatments [11]. Furthermore, it has been argued that there may be a critical 2-year period before the AD onset when inflammatory mediators are elevated [26]. Unfortunately, inflammatory mediators were not measured in the present study. On reflection it would have been of value to have ascertained whether the CIND participants had early signs of increased inflammation and if so whether they could be attenuated with omega-3 fatty acid supplements. Conversely, it may also be the case that such an intervention needs to be offered at an even earlier stage than CIND. It may be wise to undertake studies with individuals in mid to old age who display no cognitive deficits and show no evidence of elevated inflammatory mediators.

Finally, there is a large body of evidence to suggest that the appropriate omega-3 to omega-6 ratio may be the important factor to normal functioning. Even though improvements to the ratio of omega-3 fatty acids to omega-6 fatty acids through supplementation were made in this study [27], this did not seem to benefit cognition and mood in individuals with CIND and early AD. However, it has been postulated that the optimal ratio of omega-3 to 6 may vary depending on the disease of interest [28]. Reasons for this relate to the fact that diseases are multigenic and multifactorial. Therefore, therapeutic dose may depend on severity of disease taking into account possible genetic predispositions [28,29]. For that reason it may be necessary to design an intervention study that allocates different doses to individuals with varying degrees of cognitive decline. Therefore, those with CIND would be given a lower dose than those with AD.

In addition to other theoretical explanations for the present findings there are some limitations evident in this study that need consideration. Firstly, as mentioned above, the sample size was small particularly for the AD group. We acknowledge that this is a limitation. However, we believe that the findings from this study still contribute to the body of evidence (including meta-analyses) trying to determine the benefits of omega-3 fatty acids in cognition and mood in demented and non-demented individuals. In addition, there were a few measures used in this study that may have lacked the sensitivity necessary for detecting change. This was particularly true for the mood measure (BASDEC) and the BADLS. Both were subject to ceiling affects. In retrospect, the BASDEC may have been inappropriate to use with individuals with AD who lack awareness and the BADLS was inappropriate to ascertain real change in functioning in individuals with CIND. It may have been more appropriate for the study partners of the CIND participants to complete the Alzheimer’s disease Cooperative Study scale for activities of daily living in MCI (ADCS-MCI-ADL) [30], which ascertains deficits in more complex everyday tasks.

In summary, findings from this study suggest that even though omega-3 fatty acid supplementation elevates plasma concentrations of DHA and EPA in individuals with CIND and AD, this does not benefit cognition and mood in these populations. In future, studies carried out in this area should use larger sample sizes and longer study durations. Particular attention should also be paid to dose (with regards to the disease of interest, cognitive severity or dementia subtype). It is also imperative that cognitive and mood measures are chosen with caution to overcome issues surrounding sensitivity to change and ceiling and floor affects. In conclusion, at the current time it remains unclear whether the treatment of cognitive impairment and AD with omega-3 fatty acid supplements is beneficial and therefore clinically worthwhile.

## 4. Experimental Section

### 4.1. Participants

**Figure 2:**
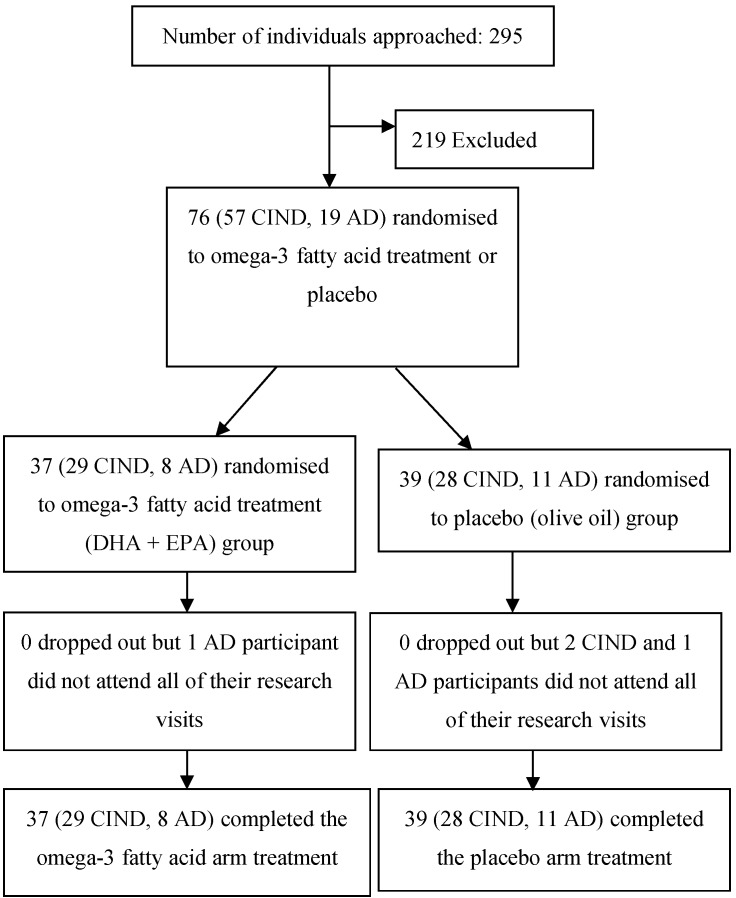
Flow diagram of progress of participants through the study.

The study was conducted between February 2006 and March 2008. In total, 76 participants (57 individuals with CIND and 19 with AD) were recruited. The patients with AD were recruited though specialist memory clinics in Bristol, UK. The CIND participants were recruited from the community via local newspaper and radio adverts. All research procedures adhered to the Helsinki Declaration (1975) and good clinical practice. Ethics approval for the study was obtained from Frenchay Research Ethics Committee, North Bristol NHS Trust on 31 October 2005 (05/Q2007/69). Informed consent was obtained from all participants as well as carer assent where necessary. Figure 2 summarises the recruitment and progress of these participants through the study. The inclusion criteria used were as follows:

AD: (1) Diagnosis based on the NINCDS-ADRDA (1984) criteria [31]; (2) Have been on a stable dose of a cholinesterase inhibitor for a minimum of three months prior to entering the study; (3) MMSE score between 16 and 30. 

CIND: (1) Self report or informant-based report of cognitive change; (2) Objective cognitive deficits falling below age-appropriate levels (Z score of −1.5); (3) An absence of dementia; (4) Retain ability to perform activities of daily living; (5) Other possible neurological or psychiatric conditions excluded. The research criteria were based on a combination of criteria for Age-associated Cognitive Decline (AACD) [32,33] criteria for all three Mild Cognitive Impairment (MCI) subtypes (amnestic MCI, multi-domain and single non-memory domain MCI).

Potential participants were excluded if (1) Another dementia was suspected, e.g., Dementia with Lewy Bodies (DLB); (2) They had a history of other psychiatric (e.g., depression (BASDEC score above ≥8), schizophrenia, *etc.*) or neurological conditions (e.g., stroke, epilepsy *etc.*); (3) They had a current drug or alcohol addiction; (4) They had disabilities, disorders or specialist dietary needs that would prevent all of the study’s requirements from being undertaken; (5) They were already taking omega-3 PUFA supplements; (6) They did not have a relative or friend that could accompany them to research visits and who lived with them or visited them at least 2 times a week.

### 4.2. Procedures and Study Design

A consecutive sampling design was used to recruit all participants to the two groups (AD and CIND). This allowed the study findings to be representative of the population from which it was drawn.

Nineteen patients (11 female, 8 male) were recruited having already had a full medical and neuropsychological examination at one of the memory clinics based in Bristol, UK. In support of their diagnosis based on the NINCDS-ADRDA (1984) criteria [31], and as part of their routine clinical care, the majority of the AD cases had already had a computed tomography or magnetic resonance image taken of their brain. Routine blood analyses were also made to discount other possible causes of their cognitive deficits. All participants came to their research appointments with a relative or friend who either lived with them or who visited them at their home on a regular basis (at least two times a week).

Fifty-seven older adults (31 female, 26 male) with a worsening memory or thinking ability were recruited from the general public through a University of Bristol press release, posters in GP surgeries as well as newspaper adverts. All 57 CIND participants were screened for suitability by the researchers using a medical questionnaire developed and based around relevant medical details obtained during diagnostic consultations at the specialist memory clinics. None of the CIND participants had previously been given a diagnosis of dementia.

Potential participants or their carer were sent two information sheets about the study, one information sheet for them and the second for their study partner (a spouse, family member or close friend who knew them well and who visited them at least twice a week during the week). After one week, they were contacted by phone to determine their interest in taking part. Participants were invited to attend either 3 (AD group) or 4 (CIND group) research appointments. The first was a screening appointment and the second, was a baseline appointment; both appointments lasted approximately one hour. The AD participants were not required to attend a screening appointment as all relevant screening documentation was gathered from their medical records once they had been approached to take part in the study by their treatment team. The last two appointments, which measured performance change from baseline took approximately one and a half to two hours each. The participants took part in the study for 4 months.

Participants were randomised to receive two capsules containing a total of 625 mg DHA and 600 mg EPA, or two placebo capsules containing olive oil each day for four months. Each capsule (EPA + DHA and placebo) also contained 10 mg mixed tocopherols. Participants were requested to take the capsules at a convenient time of day for them, preferably with food. This information, as well as their participant ID, visit number and date of visit was also printed on a label attached to the outside of each container. Participants and their carers and the researchers conducting the cognitive assessments and the plasma fatty-acid assays were blind to the identity of the treatments (EPA + DHA or placebo).

At baseline, month 1 and month 4 the following examinations were completed: fasting bloods, assessment of functioning in daily living, cognition, mood and visual attention. Blood samples were taken to assess compliance with the intervention. This, the fatty acid profile of plasma phosphatidylcholine (PC) was analysed by gas chromatography. Total lipid was extracted from plasma using chloroform-methanol (2:1 *v*/*v*). PC was isolated by solid phase extraction. Fatty acid methyl esters were prepared by incubation of purified plasma PC with methanol in sulphuric acid and were separated using a Hewlett Packard 6890 gas chromatograph (Agilent, Cheshire, UK) equipped with a 30 m × 0.25 µm × 0.25 mm BPX-70 fused silica capillary column (SGE, Milton Keynes, UK) and flame ionisation detection. The concentrations of individual fatty acids in plasma PC were determined by measurement of the peak area using ChemStation software (Agilent) and each fatty acid was expressed as a proportion of the total [34]. Calculations were based on percentage contribution each fatty acid makes to the total fatty acids identified in plasma PC. In total, 25 fatty acids were identified which accounted for 99% of the peaks found in a human plasma PC gas chromatography trace.

All participants self-reported that they had normal or corrected to normal vision and hearing.

### 4.3. Consent

Consent from both the participant and their study partner was obtained at the screening appointment or at the baseline appointment for the AD group. Copies of the consent and assent forms were given to the participants and study partners to remind them of their role and their rights to withdraw. Furthermore, a copy of the consent forms for the AD participants was also filed with their medical records. This was an ethical requirement under the terms of the project’s research ethics application.

### 4.4. Outcome Measures

The primary outcome variables used within this study were the MMSE [35], the Hopkins Verbal Learning Test—Revised (HVLT-R) [36] and the Brief Assessment Schedule Depression Cards (BASDEC) [37]. The secondary outcome variables were other neurospsychological measures such as tests looking at executive functioning [38], language [39], verbal reasoning [40], visual memory [41], as well as the Bristol’s Activities of Daily Living Scale (BADLS) [42].

### 4.5. Statistical Analysis

Two types of analysis were conducted; intention to treat (using the last observation carried forward (LOCF) method) and per-protocol. Very few differences in results were observed between the two analyses, therefore the data presented here represent the LOCF method unless otherwise stated.

Longitudinal changes in the outcome variables were analysed using repeated measures analysis of variance. The data are shown as means, standard deviations, and 95% confidence intervals.

### 4.6. Ethical Considerations

This study abided by all the appropriate ethical guidelines outlined by the Declaration of Helsinki and was undertaken according to good clinical practice. Informed consent was obtained from all participants and assent obtained from their study partner. This study was approved by the Local Ethics Committee of North Bristol NHS Trust, Bristol UK and approved by the Research and Development departments of Avon and Wiltshire Mental Health Partnership Trust (AWP) and Bristol Primary Care Trust. The sponsor of the study was the University of Bristol.

## References

[1] Lunn J., Theobald H.E. (2006). The health effects of dietary unsaturated fatty acids. Nutr. Bull..

[2] Iso H., Rexrode K.M., Stampfer M.J., Manson J.E., Colditz G.A., Speizer F.E., Hennekens C.H., Willett W.C. (2001). Intake of fish and omega-3 fatty acids and risk of stroke in women. JAMA.

[3] Virtanen J.K., Siscovick D.S., Longstreth W.T., Kuller L.H., Mozaffarian D. (2008). Fish consumption and risk of subclinical brain abnormalities on MRI in older adults. Neurology.

[4] Hornstra G. (2001). Influence of dietary fat type on arterial thrombosis tendency. J. Nutr. Health Aging.

[5] Von Schacky C., Harris W.S. (2007). Cardiovascular benefits of omega-3 fatty acids. Cardiovasc. Res..

[6] Sydenham E., Dangour A.D., Lim W.S. (2012). Omega-3 fatty acid for the prevention of cognitive decline and dementia. Cochrane Database Syst. Rev..

[7] Vakhapova V., Cohen T., Richter Y., Herzog Y., Korczyn A.D. (2010). Phosphatidylserine containing omega-3 fatty acids may improve memory abilities in non-demented elderly with memory complaints: A double-blind placebo-controlled trial. Dement. Geriatr. Cogn. Disord..

[8] Otsuka M. (2000). Analysis of dietary factors in Alzheimer’s disease: Clinical use of nutritional intervention for prevention and treatment of dementia. Nippon Ronen Igakkai Zasshi.

[9] Yehuda S., Rabinovtz S., Carasso R.L., Mostofsky D.I. (1996). Essential fatty acids preparation (SR-3) improves Alzheimer’s patients quality of life. Int. J. Neurosci..

[10] Boston P.F., Bennett A., Horrobin D.F., Bennett C.N. (2004). Ethyl-EPA in Alzheimer’s disease—A pilot study. Prostaglandins Leukot. Essent. Fatty Acids.

[11] Freund-Levi Y., Eriksdotter-Jönhagen M., Cederholm T., Basun H., Faxén-Irving G., Garlind A., Vedin I., Vessby B., Wahlund L.-O., Palmblad J. (2006). Omega-3 fatty acid treatment in 174 patients with mild to moderate Alzheimer disease: OmegAD study: A randomized double-blind trial. Arch. Neurol..

[12] Freund-Levi Y., Basun H., Cederholm T., Faxén-Irving G., Garlind A., Grut M., Vedin I., Palmblad J., Wahlund L.-O., Eriksdotter-Jönhagen M. (2008). Omega-3 supplementation in mild to moderate Alzheimer’s disease: Effects on neuropsychiatric symptoms. Int. J. Geriatr. Psychiatry.

[13] Appleton K.M., Hayward R.C., Gunnell D., Peters T.J., Rogers P.J., Kessler D., Ness A.R. (2006). Effects of n-3 long-chain polyunsaturated fatty acids on depressed mood: Systematic review of published trials. Am. J. Clin. Nutr..

[14] Kontani S., Sakaguchi E., Warashina S., Matsukawa N., Ishikura Y., Kiso Y., Sakakibara M., Yoshimoto T., Guo J., Yamashima T. (2006). Dietary supplementation of arachidonic and docosahexaenoic acids improves cognitive dysfunction. Neurosci. Res..

[15] Chiu C.C., Su K.P., Cheng T.C., Lui H.C., Chang C.J., Dewey M.E., Stewart R., Huang S.Y. (2008). The effects of Omega-3 fatty acids monotherapy in Alzheimer’s Disease and Mild Cognitive Impairment: A preliminary randomized double blind placebo controlled study. Prog. Neuro-Psychopharmacol..

[16] Dangour A.D., Allen E., Elbourne D., Fasey N., Fletcher A.E., Hardy P., Holder G.E., Knight R., Letley L., Richards M. (2010). Effect of 2-y n-3 long-chain polyunsaturated fatty acid supplementation on cognitive function in older people: A randomized, double-blind, controlled trial. Am. J. Clin. Nutr..

[17] Van de Rest O., Geleijnse J.M., Kok F.J., van Staveren W.A., Hoefnagels W.H., Beekman A.T.F., de Groot L.C.P.G.M. (2008). Effects of fish-oil supplementation on mental well-being in older subjects: A randomised, double-blind, placebo-controlled trial. Am. J. Clin. Nutr..

[18] Van de Rest O., Geleijnse J.M., Kok F.J., van Staveren W.A., Dullemeijer C., OldeRikkert M.G.M., Beekman A.T.F., de Groot C.P.G.M. (2008). Effect of fish oil on cognitive performance in older subjects. Neurology.

[19] Geleijnse J.M., Giltay E.J., Kromhout D. (2012). Effects of n-3 fatty acids on cognitive decline: A randomized, double-blind, placebo-controlled trial in stable myocardial infarction patients. Alzheimers Dement..

[20] Nelson H.E., Willison J.R. (1991). National Adult Reading Test (NART). Test Manual Including New Data Supplement.

[21] Terano T., Fujishiro S., Ban T., Yamamoto K., Tanaka T., Noguchi Y., Tamura Y., Yazawa K., Hirayama T. (1999). Docosahexaenoic acid supplementation improves the moderately severe dementia from thrombotic cerebrovascular diseases. Lipids.

[22] Grosso G., Pajak A., Marventano S., Castellano S., Bucolo C., Drago F., Caraci F. (2014). Role of omega-3 fatty acids in the treatment of depressive disorders: A comprehensive meta-analysis of randomized clinical trials. PLoS ONE.

[23] Browning L.M., Walker C.G., Mander A.P., West A.L., Madden J., Gambell J.M., Young S., Wang L., Jebb S.A., Calder P.C. (2012). Incorporation of eicosapentaenoic and docosahexaenoic acids into lipid pools when given as supplements providing doses equivalent to typical intakes of oily fish. Am. J. Clin. Nutr..

[24] Bascoul C. (2007). How can omega-3 fatty acids prevent Alzheimer’s disease?.

[25] Newman P.E. (2000). Alzheimer’s disease revisited. Med. Hypotheses.

[26] In ’t Veld B.A., Ruitenberg A., Hofman A., Launer L.J., van Duijn C.K., Stinjen T., Breteler M.B.B., Stricker B.H.C. (2001). Nonsteroidal antiinflammatory drugs and the risk of Alzheimer’s disease. N. Engl. J. Med..

[27] Phillips M., Childs C., Calder P., Rogers P. (2012). Lower omega-3 fatty acid intake and status are associated with poorer cognitive function in older age: A comparision of individuals with and without cognitive impairment and Alzheimer’s disease. Nutr. Neurosci..

[28] Simopoulos A.P. (2002). The importance of the ratio of omega-6/omega-3 essential fatty acids. Biomed. Pharmacother..

[29] Simopoulos A.P. (2002). Omega-3 Fatty acids in inflammation and autoimmune diseases. J. Am. Coll. Nutr..

[30] Pedrosa H., de Sa A., Guerreiro M., Maroco J., Simoes M.R., Galasko D., de Mendonca A. (2010). Functional evaluation distinguishes MCI patients from healthy elderly people—The ADCS/MCI/ADL scale. J. Nutr. Health Aging.

[31] McKhann G., Drachman D., Folstein M., Katzman R., Price D., Stadlan E.M. (1984). Clinical diagnosis of Alzheimer’s disease: Report of the NINCDS-ADRDA work group* under the auspoces of department of health and human services task force on Alzheimer’s disease. Neurology.

[32] Levy R. (1994). Aging associated cognitive decline. Int. Psychogeriatr..

[33] Petersen R.C. (2004). Mild cognitive impairment as a diagnostic entity. Intern. Med..

[34] Fisk H.L., West A.L., Childs C.E., Burdge G.C., Calder P.C. (2014). The use of gas chromatography to analyze compositional changes of fatty acids in rat liver tissue during pregnancy. J. Vis. Exp..

[35] Folstein M.F., Folstein S.E., McHugh P.R. (1975). “Mini-mental State”: A practical method for grading the cognitive state of patients for the clinician. J. Psychiatr. Res..

[36] Brandt J. (1991). The Hopkins Verbal Learning Test: Development of a new memory test with six equivalent forms. Clin. Neuropsychol..

[37] Adshead F., Cody D.D., Pitt B. (1992). BASDEC: A novel screening instrument for depression in elderly medical in-patients. Br. Med. J..

[38] Royall D.R., Cordes J.A., Polk M. (1998). CLOX: An executive clock drawing task. J. Neurol. Neurosur. Psychiatry.

[39] Kaplan E., Goodglass H., Weintraub S. (2001). Boston Naming Test.

[40] Wechsler A. (1999). Wechsler Adult Intelligence Scale.

[41] Wilson B.A., Clare L., Baddeley A., Cockburn J., Watson P., Tate R. (1998). The Rivermead Behavioural Memory Test—Extended Version.

[42] Bucks R.S., Ashworth D.L., Wilcock G.K., Siegfried K. (1996). Assessment of activities of daily living in dementia: Development of the Bristol activities of daily living scale. Age Ageing.

